# Contingent Mother’s Voice Intervention Targeting Feeding in Hospitalized Infants with Critical Congenital Heart Defects

**DOI:** 10.3390/children10101642

**Published:** 2023-09-30

**Authors:** Caitlin P. Kjeldsen, Lelia Emery, Janet Simsic, Zhulin He, Ann R. Stark, Mary Lauren Neel, Nathalie L. Maitre

**Affiliations:** 1 Department of Speech and Hearing Science, The Ohio State University, Columbus, OH 43210, USA; ckjelds@emory.edu; 2 School of Medicine and Children’s Healthcare of Atlanta, Emory University, Atlanta, GA 30306, USA; 3 Nationwide Children’s Hospital, Columbus, OH 43205, USA; 4 Beth Israel Deaconess Medical Center, Department of Neonatology, Boston, MA 02215, USA

**Keywords:** mother’s voice, critical congenital heart defects, CCHD, neurodevelopment, feeding, non-nutritive sucking, music therapy

## Abstract

Infants with critical congenital heart defects (CCHD) are at high risk for feeding challenges and neurodevelopmental delays; however, few interventions promoting the neurodevelopmental progression of feeding have been studied with this population. Contingent mother’s voice has been successfully used as positive reinforcement for non-nutritive suck (NNS) in studies with preterm infants, leading to improved weight gain and more rapid cessation of tube feedings; however, this type of intervention has not been studied in infants with CCHD. This study aimed to determine whether an NNS-training protocol using the mother’s voice as positive reinforcement and validated in preterm infants could improve oral feeding outcomes in hospitalized infants with CCHD undergoing cardiac surgical procedures. Infants were randomized to receive the contingent mother’s voice intervention before or after cardiac surgery, with a control comparison group receiving passive exposure to the mother’s voice after surgery. There were no significant differences in discharge weight, PO intake, length of stay, time to full feeds, or feeding status at 1-month post-discharge between infants who received contingent mother’s voice compared to those who did not. There were significant differences in PO intake and time to full feeds following surgery based on infants’ pre-enrollment PO status and severity of illness. At 1-month post-discharge, parents of infants in the intervention group expressed a higher rate of positive feelings and fewer concerns regarding their infant’s feeding compared to parents of infants in the control group. While the current protocol of 5 sessions was not associated with improved feeding outcomes in infants with CCHD, it empowered parents to contribute to their infant’s care and demonstrated the feasibility of using the mother’s voice as positive reinforcement for infants with CCHD. Further study of timing, intensity, and duration of interventions leveraging the mother’s voice in this population is needed. ClinicalTrials.gov Identifier: NCT03035552.

## 1. Introduction

Congenital heart defects (CHD) account for >30% of all congenital anomalies each year [1], and one-quarter of infants with critical congenital heart defects (CCHD) require surgical intervention early in life [2]. Affected infants often have neurodevelopmental delays with high rates of impaired oral feeding skills, poor growth, and poor motor and language outcomes [3,4,5]. In addition, poor feeding and failure to grow in the first year of life are associated with increased mortality following cardiac surgery [6,7]. While overall mortality among children with CHD has declined in recent years, the incidence of neurodevelopmental problems has increased [8], and early neurodevelopmental impairment is associated with learning difficulties, executive function disorders, and poor social-emotional adaptation in adolescence and adulthood [9,10].

Recent calls for increased attention to the neurodevelopmental needs of hospitalized infants with CCHD have highlighted the need for a better understanding of how early hospitalization and the critical care experience impact long-term development [11,12]. During hospitalization, infants with CCHD are frequently separated from their parents and exposed to atypical sensory stimulation, such as high levels of environmental noise and limited opportunities for out-of-bed holding [13,14]. In addition to infants’ needs, parents of infants with CCHD often experience high levels of stress, anxiety, and depression related to their infant’s diagnosis and care [15]. The COVID-19 pandemic and its associated disruptions further exacerbated the challenges parents face when their children are hospitalized; parents reported feeling isolated and disconnected from their hospitalized children, especially when the parental role of caregiver was restricted [16]. The combined needs of infants with CCHD and their parents necessitate the study of innovative, developmentally supportive interventions that are feasible and safe during this sensitive period of development [11].

Currently, few evidence-based oral feeding interventions exist for infants with CCHD during their initial hospitalization. Most available protocols aim to increase feeding volume to support optimal weight gain and minimize the incidence of necrotizing enterocolitis (NEC) [17,18,19,20] rather than to promote a neurodevelopmental progression of feeding competence [21]. Further, no consensus exists on when and how to begin feeding, and feeding practices vary considerably across institutions [22]. Recent expanded attention to developmental care in pediatric and cardiac intensive care has highlighted the need for supportive interventions to promote short- and long-term neurodevelopment in these infants and children [8,23,24].

Interventions supporting the neurodevelopmental progression of feeding skills in preterm infants have been shown to be safe and effective [25]; however, they have not yet been reported in infants with CCHD. Interventions utilizing a pacifier-activated music (PAM) player that rely on the principles of operant conditioning with positive reinforcement to establish and strengthen functional pathways between lower- and higher-order neural networks before maladaptive patterns occur may have potential with this population. This non-nutritive suck (NNS)-contingent training approach has been shown to improve preterm infant behavioral state regulation, promote sucking behavior, improve transitions from enteral to oral feedings [25,26,27,28], and increase preterm infants’ speech-sound differentiation ability [29]. Suck-contingent mother’s voice, specifically, has been shown to effectively improve oral feeding coordination and rate and is associated with the acquisition of essential developmental milestones in the first year of life [25].

Because NNS training may provide a low-cost and developmentally appropriate way to promote feeding in infants with CCHD, we tested the hypothesis that an intensive NNS training protocol using the positive reinforcement of the mother’s voice would improve oral feeding outcomes in infants with CCHD by improving the strength of NNS.

## 2. Methods

Our original study design was a prospective randomized controlled trial with waitlisted controls, in which one group of infants received the intervention, NNS-contingent mother’s voice, before surgery while the waitlisted group received the intervention after surgery. The study was powered to recruit 34 patients per group. However, following IRB approval and study initiation, new enrollment restrictions in the Cardiothoracic Intensive Care Unit (CTICU) prevented access to infants with single ventricle defects such as Hypoplastic Left Heart Syndrome, substantially decreasing the available population. To account for the decreased number of eligible infants, we modified the study design to add a third group that received only passive mother’s voice. The study and modifications were approved by the Institutional Review Board at Nationwide Children’s Hospital, and informed consent was obtained from all subjects’ parents prior to enrollment (IRB16-00493). The study was registered at ClinicalTrials.gov (ClinicalTrials.gov Identifier: NCT03035552).

We recruited infants from the Neonatal Intensive Care Unit (NICU) and the CTICU if they had a diagnosis of a critical congenital heart defect, were scheduled for surgical intervention, had a postmenstrual age (PMA) >37 weeks at study start and were taking less than 50% of their nutrition by mouth (PO). Infants with acquired brain injury were included. Infants were excluded if, at the time of the study, they required respiratory support with assisted ventilation, had received general anesthesia within 24 h, or had lethal congenital abnormalities.

Following informed consent, mothers were recorded singing native language lullabies using a Sony PCM-M10 Portable Linear Voice Recorder (Sony, New York, NY, USA). Infants were randomized using random-block allocation to one of three groups: pre-surgery contingent mother’s voice, post-surgery contingent mother’s voice, or passive mother’s voice only. The intervention was delivered via a pacifier-activated music (PAM) player (PAL, Powers Device Technologies, Boca Raton, FL, USA), a device that measures the timing and air displacement of an infant’s suck via a sensor inserted into a standard pacifier. The study used the protocol reported in a randomized controlled trial of a feeding intervention for preterm infants [25]. When the infant achieved the predetermined suck threshold, the mother’s voice played for 10 s. After 10 s, the music would stop, and the infant would again be required to meet the predetermined settings to re-activate the mother’s voice. The study therapist modulated the settings of the PAM according to our IRB-approved protocol.

The intervention consisted of five 15-minute PAM sessions, 1–2 times per day. The control infants received five 15-minute passive listening sessions of their mother’s voice recording using the non-contingent setting on the PAM, and non-nutritive sucking (NNS) was assessed with no voice reinforcement. For all groups, in the event of a bradycardia/desaturation episode during a session, the session was paused, and stimulation was provided per unit protocol. If the infant recovered rapidly, the session resumed. In the event of a second bradycardia/desaturation episode, the session was concluded and reattempted at a different time.

NNS data collected during pre- and post-assessments included the number of sucks in a suck burst, pause time between suck bursts, and average air displacement threshold of sucks in a burst (suck strength). Additional data collected were weight, PO status (oral versus enteral versus intravenous), and PO volume at consent and 24 h after the final assessment; number of days from birth to full PO feeds; number of days from surgery to full PO feeds; and feeding status at discharge. Parents were asked to complete a questionnaire about feeding assessments at 1-month post-discharge. Although the initial study design included a 12-month follow-up for feeding and neurodevelopmental assessments, this was not accomplished due to high mortality rates and lower-than-expected enrollment.

### Statistical Analyses

Primary proximal outcomes included feeding measures in the NICU/CTICU (weight gain, PO status, PO volume) and at discharge (number of days to full feeds, feeding status). Distal outcomes included feeding status and enrollment in feeding therapy at 1-month after discharge, as well as qualitative feedback on feeding from parents. All data on hospital stay, presence of a feeding tube, and growth were obtained from the electronic medical record; one-month feeding status and parental perception of feeding ability was completed by parents via phone questionnaire.

Infants’ risk of mortality was assessed following the Society of Thoracic Surgeons-European Association for Cardio-Thoracic Surgery (STAT) scoring tool, a tool designed to analyze the risk for mortality associated with congenital heart surgery procedures [30]. Categories 1 through 5 were assigned to subjects, with 1 indicating lowest and 5 indicating highest risk for mortality.

Outcomes were assessed using logistic regression for categorical variables and generalized linear models for continuous variables. STAT category was included as a covariate in all analyses due to its potential impact on outcomes. All analyses were conducted using the R Studio statistical program, version 4.2.0 (R Core Team, Vienna, Austria), with two-sided *p*-values < 0.05 considered statistically significant.

## 3. Results

**Figure 1:**
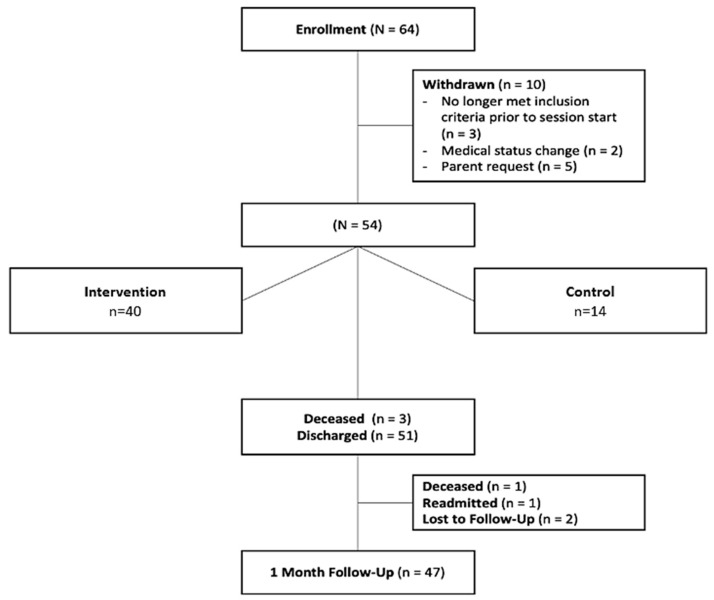
Participants.

The initial enrollment goal was 72 infants; however, recruitment restrictions in the CTICU following study initiation and IRB approval resulted in lower-than-expected numbers of eligible infants to achieve this goal. In addition, research restrictions due to the COVID-19 pandemic resulted in early termination of the study. Sixty-four infants were enrolled in total. Ten subjects were withdrawn prior to the completion of inpatient sessions. (Figure 1).

Fifty-four subjects completed all inpatient sessions. Three subjects died before hospital discharge, and one died shortly after discharge, before the 1-month follow-up. Of the 50 remaining subjects, 47 completed follow-up at one month post-discharge (94%); one subject was readmitted to the hospital and unable to attend the follow-up visit, and two were lost to follow-up.

Patient demographics were analyzed by Fisher’s exact and Kruskal–Wallis rank sum tests (Table 1).

Sex, gestational age, and race were not statistically different between groups. Group sizes were considerably different given the addition of the non-waitlist control after imposed research restrictions, with a larger number of subjects receiving the intervention (*n* = 40) compared to those receiving only control (*n* = 14). Nutrition status at pre-assessment differed significantly between groups (*p* = 0.007), with 3/14 infants in the control group primarily receiving nutrition PO compared to 0/40 in the intervention group; these infants remained eligible for the study because they were also receiving a combination of enteral (feeding tube) and TPN nutrition, and thus their PO intake accounted for less than 50% of their overall nutrition. As a result, we included primary nutrition status at pre-assessment as a covariate with categories including TPN, fully enteral, or partial PO. Additionally, although not statistically significant between groups, six patients in the intervention group had abnormal neuroimaging findings compared to none in the control group.

### 3.1. Quantitative Outcomes

Primary outcomes are shown in Table 2a,b. Based on primary nutrition status (oral versus enteral versus intravenous), oral intake changed significantly, increasing 24% with each change in category in pre-assessment oral intake status.

Feeding outcomes were evaluated at hospital discharge (Table 3) and one-month post-discharge. Feeding outcomes at one-month post-discharge were not different, likely due to small numbers (Table 4). At discharge, the primary feeding method at pre-assessment was a significant predictor of number of days from birth to full feeds and from surgery to full feeds. STAT category was also a significant predictor of number of days from surgery to full feeds, with an increase of 53% for one level higher of the STAT category. Although not significantly different, the number of days to achieve full feeds following surgery appeared 43% lower for the intervention group compared to the control group. The number of subjects discharged with a G-Tube and the number feeding fully PO at discharge did not differ between groups.

### 3.2. Qualitative Outcomes

Parent perceptions of infant feeding skills were assessed through two open-ended questions at one month post-discharge: (1) How is feeding going in your opinion? and (2) Do you have any feeding concerns? Parents of twenty-two out of thirty-five infants (63%) in the intervention group completed the 1-month follow-up feeding questionnaire. Eight out of twelve parents (67%) completed the 1-month follow-up feeding questionnaire in the control group. Parent responses are presented in Table 5. Even with uneven samples in each group, parents of infants in the control group reported more concerns with feeding than parents of infants in the intervention group.

## 4. Discussion

We report for the first time the application of NNS training using reinforcement with the mother’s voice in infants with CCHD requiring surgical repair. Although this intervention has been shown to improve oral feeding skills in preterm infants [25], our use of the same protocol did not demonstrate similar results in older infants with CCHD, who had no significant improvements in feeding outcomes during hospitalization or at one month post-discharge. Modifications in study design, uneven group sizes, small sample size, and the significant difference in primary feeding status at pre-assessment may have contributed to these results.

Both the method of primary nutrition and STAT category at pre-assessment were significant predictors of the number of days from birth to full feeds and from surgery to full feeds. In infants who fed PO prior to surgery, time to full feeds was 73% less after surgery compared to those who did not. We also saw a 53% increase in time to achieve full PO feeds for each increasing level in the STAT category, likely corresponding to the increasing risk for mortality and morbidity and, thus, severity of illness.

We observed a trend of 43% fewer days to achieve full feeds following surgery in the intervention group compared to the control, although no differences were seen between groups in number of infants feeding fully PO or with a G-tube. Statistical significance was limited by the small sample size. An additional limitation was the difference in primary nutrition status between the groups related to three control infants who received the largest proportion of their nutrition PO pre-enrollment. Although these infants met the stated inclusion criteria because their PO volume accounted for less than 50% of the combined parenteral nutrition/enteral volume, given the overall small numbers, this may have resulted in a type II error.

While previous work has shown that efficient NNS is not entirely predictive of later oral feeding abilities, NNS training allows for the safe reinforcement of coordinated suck-burst patterns without the added element of fluid management. This type of intervention is safe for infants with dysphagia or oro-sensory aversions as it provides an opportunity for NNS practice without the simultaneous introduction of oral liquid [25]. Given the incidence of laryngopharyngeal dysfunction in infants with CCHD, this type of intervention may be considered particularly favorable for addressing suck-specific concerns [31].

While the current protocol did not result in improved feeding outcomes, two new hypotheses emerged. First, the mechanism underlying feeding impairment in infants with CCHD may differ from that in preterm infants, thus limiting the effect of a suck-focused intervention. Secondly, the current protocol of five sessions may not have provided sufficient practice with reinforced NNS to impact feeding. Further investigation of how NNS training may reinforce coordinated suck-burst patterns with a distinction between infants whose feeding difficulties are or are not related to dysphagia or laryngopharyngeal dysfunction is needed.

Though feeding was the primary outcome of interest for this particular study, an essential component was the use of the mother’s voice as the reinforcing stimulus. As the mother’s voice is the most salient auditory stimulus fetuses experience in utero [32], exposure to the mother’s voice recording during hospitalization is considered developmentally appropriate and has previously been shown to be effective when used as a positive behavioral reinforcer after birth [25,33,34]. In addition, neuroscience-based work with hospitalized infants has shown that infants demonstrate an immediate and positive cortical response to their mother’s voice, a response that is seen even in immature infants [35]; this response is decreased when infants are exposed to other female voices instead [36]. While the present intervention had minimal impact on feeding, the exposure to the mother’s voice was still able to provide the infants with developmentally appropriate language exposure in an easily accessible and low-cost way.

In addition to addressing the developmental needs of the infants, this intervention provided mothers with a unique opportunity to contribute to and engage in their infant’s care in a way only they could—by providing a recording of their voice. By empowering mothers to actively participate in their infant’s treatment, it is possible that mothers’ self-efficacy, attachment, and bonding were improved. Previous work has shown that higher maternal self-efficacy is associated with healthier mother–child feeding practices later in childhood [37], and greater maternal bonding is a potentially modifiable predictor of infant outcomes [38]. It is possible the qualitative differences in feeding reported by parents after discharge were related to improved maternal self-efficacy and/or attachment. This consequently may have left parents feeling more equipped to manage feeding problems long-term.

### Implications

This intervention relies on parental voice, is developmentally supportive, and can easily be implemented in intensive care units even when parents have barriers to frequent visitation. While the current intervention did not result in improved feeding outcomes, additional research on intervention intensity and frequency is warranted. Importantly, parent perceptions of their infant’s feeding may improve with this intervention; providing exposure to the mother’s voice recording may be mutually beneficial for infants and mothers by empowering parents to contribute to their infant’s care and may lead to improved self-efficacy in caring for their infant.

## 5. Conclusions

We have demonstrated the feasibility of implementing a short-term, parent-driven study in infants with CCHD that leverages the mother’s voice and may improve feeding outcomes in this high-risk infant population. Although our study was limited by small sample size and protocol modifications due to unit research restrictions as well as the COVID-19 pandemic and resultant research restrictions, future adequately powered studies promoting oral feeding with NNS-contingent mother’s voice, a successful intervention in preterm infants, are warranted in this and other high-risk populations.

## Figures and Tables

**Table 1:** Patient Demographic Characteristics (N = 54).

	Intervention (N = 40) ^1^	Control (N = 14) ^1^	*p*-Value ^2^
**Deceased**	6 (15)	3 (21)	0.427
**Sex**			
Female	23 (58)	5 (36)	0.137
Male	17 (42)	9 (64)	
**Gestational Age at Birth**	39.0 (36.75–39.0)	37.5 (35.5–39.0)	0.281
**Birthweight**	3089.5 (2510–3515)	3040 (2506.25–3528.25)	0.737
**Race**			0.182
White	29 (72)	8 (57)	
Black/African American	5 (13)	5 (36)	
Native Hawaiian or Pacific Islander	0 (0)	0 (0)	
Asian	4 (10)	0 (0)	
American Indian or Alaska Native	0 (0)	0 (0)	
Other	2 (5)	1 (7)	
**Presence of Neural Insult Diagnosis**	6 (11)	0 (0)	0.129
**Type of Neural Insult ^3^**			0.462
Perinatal hypoxia	1 (3)	0 (0)	
IVH	2 (5)	0 (0)	
Other (malformation)	3 (7)	0 (0)	
None	31 (78)	14 (100)	
Unknown	3 (7)	0 (0)	
**STAT Category ^4^**			0.676
STAT 1	5 (13)	0 (0)	
STAT 2	13 (32)	5 (36)	
STAT 3	5 (13)	2 (14)	
STAT 4	14 (35)	5 (36)	
STAT 5	3 (7)	2 (14)	
**Primary Source of Nutrition at Pre-Assessment ^5,6^**			**0.007**
PO	0 (0)	3 (21)	
TPN	31 (78)	7 (50)	
NG/OG	9 (22)	4 (29)	
G-tube	0 (0)	0 (0)	

^1^ Median (IQR); n (%). ^2^ Fisher’s exact and Kruskal–Wallis rank sum tests. ^3^ Neural insult diagnosis defined as intraventricular hemorrhage grades III or IV, perinatal hypoxia, periventricular leukomalacia, diffuse ischemia, or agenesis of the corpus callosum; all findings documented on magnetic resonance imaging. ^4^ STAT Category—Infant’s risk of mortality as defined by the Society of Thoracic Surgeons-European Association for Cardio-Thoracic Surgery (STAT) scoring tool. ^5^ Infants receiving IV or enteral nutrition may have also been taking some PO; however, this indicates their primary source of nutrition. ^6^ Infants coded as primarily PO nutrition at the pre-assessment were also receiving a combination of enteral/TPN nutrition, leading to their PO intake accounting for less than 50% of their total nutrition and therefore not meeting exclusion criteria.

**Table 2:** (a). Primary Source of Nutrition Pre- and Post-Study Participation in NICU/CTICU. (b). Feeding Outcomes in NICU/CTICU (N = 54).

(a)							
**Feeding Status**	**Intervention, N = 40 ^1^**		**Control, N = 14 ^1^**				
	**PRE**	**POST**	**PRE**	**POST**			
**Oral**	0 (0)	11 (28)	**3 (21)**	4 (29)			
TPN *	31 (78)	9 (22)	7 (50)	1 (7)			
**NG/OG**	9 (22)	17 (43)	4 (29)	9 (64)			
**G-Tube**	0 (0)	3 (7)	0 (0)	0 (0)			
^1^ n (%).							
(b)							
**Feeding Outcomes**	**Intervention, N = 40 ^1^**		**Control, N = 14 ^1^**		**Beta**	**95% CI**	***p*** **-Value ^2^**
**Percentage Weight Change**	2.090 (−2.301, 7.524)		2.293 (−1.011, 7.117)				
Intervention					0.03	−0.09, 0.14	0.7
STAT Category					−0.02	−0.06, 0.02	0.3
Primary Nutrition at Pre-Assessment					−0.01	−0.10, 0.07	0.8
**Change in Percentage PO Intake**	9.169 (0.00, 87.456)		29.110 (0.00, 48.113)				
Intervention					−0.07	−0.36, 0.22	0.6
STAT Category					−0.04	−0.14, 0.06	0.4
Primary Nutrition at Pre-Assessment					0.24	0.02, 0.46	**0.037**

^1^ Median (IQR). ^2^ Gaussian Generalized Linear Model with identify link function used for continuous variables. Covariates included primary nutrition status at pre-assessment and STAT category.

**Table 3:** Feeding Outcomes at Discharge (N = 51).

At Discharge (Minus Deceased)	Intervention N = 39 ^1^	Control N = 12 ^1^	IRR	95% CI	*p*-Value ^2^
**Days from Birth to Full Feeds**	33 (27, 56)	41 (26, 109)			
Intervention			1.16	0.57, 2.26	0.7
STAT Category			1.06	0.82, 1.36	0.6
Primary Nutrition at Pre-Assessment			0.42	0.23, 0.73	**<0.001**
**Days from Surgery to Full Feeds**	8 (4, 16)	26 (8, 43)			
Intervention			0.57	0.21, 1.39	0.2
STAT Category			1.53	1.09, 2.13	**0.012**
Primary Nutrition at Pre-Assessment			0.27	0.12, 0.60	**<0.001**
**Discharged with G-Tube**	3 (8)	0 (0)			0.631
**Full PO at Discharge**	17 (44)	8 (67)			0.488

^1^ Median (IQR); n (%). ^2^ Negative Binomial Regression with log link used for continuous variables. Logistic regression used for categorical variables. Covariates included primary nutrition status at pre-assessment and STAT category.

**Table 4:** Feeding Outcomes at 1-month post-discharge; N = 47.

Characteristic	Intervention, N = 35 ^1^	Control, N = 12 ^1^	*p*-Value ^2^
**Completely PO at 1 Month**	18 (51)	8 (67)	0.438
Presence of Feeding Tube	17 (49)	4 (33)	
**Completely Enteral**	6 (17)	2 (17)	0.254
**Enrolled in Feeding Therapy**			0.461
Enrolled	11 (31)	1 (8)	
Not enrolled	22 (63)	9 (75)	
Unknown	2 (6)	2 (17)	
Only Those PO at 1 Month FU	N = 29	N = 10	*p*-value ^2^
**Time to PO Feed**			0.769
<30 min	24 (83)	8 (80)	
>30 min	3 (10)	2 (20)	
Unknown	2 (7)	0 (0)	
**Volume per Feed**			
<4 oz.	20 (69)	9 (90)	0.148
>4 oz.	8 (28)	1 (10)	
Unknown	1 (3)	0 (0)	

^1^ n (%). ^2^ Logistic regression used for categorical variables. Generalized Linear Model with log link function used for continuous variables. Covariates included primary nutrition status at pre-assessment and STAT category.

**Table 5:** Qualitative Report on Feeding from Parents at One-Month Post-Discharge.

	Intervention (*n* = 22/35)	Control (*n* = 8/12)
Parent reported concerns with feeding *	Feeding is “not [going] well.” The infant “chokes a lot and only takes minimal PO.” Feeding is “okay,” but the infant “sounds hoarse after he eats and will gag and cough while eating at times.” Family requested video swallow study and feeding therapy through the hospital but has not yet received a response	“Honestly, feeding has been pretty frustrating.” Frustration with feeding due to frequent stiffening and arching. Parent submitted request for outpatient feeding assessment that has not yet occurred Infant had a backslide after another procedure and is making slow progress returning to baseline Infant sucks on pacifier during G-tube feeds as it is not safe for her to feed PO. [Mother] worried about baby coughing. A repeat swallow study is planned for the future. Infant is not enrolled in feeding therapy or followed by feeding specialist
Parent reported positive feelings about feeding *	Feeding is “enjoyable now” Feeding is “awesome. She’s eating like a champ.” Feeding is “going really well since her last surgery.” The infant “does really good with sucking, swallowing, and breathing.” Feeding is going “really well,” and the infant is “doing awesome.” Feeding is “going well” and the infant is “more active, not exhausted like before surgery.” Infant “gets excited when she sees the baby food jar.”	Feeding is “going just fine, very normal.” “So far, so good.”

* Direct quotes provided in quotation marks.

## Data Availability

Data are available on request due to privacy restrictions.

## References

[1] Critical Congenital Heart Defects in the United States Centers for Disease Control and Prevention. Updated 24 January 2022. https://www.cdc.gov/ncbddd/heartdefects/features/cchd-keyfindings.html#:~:text=Congenital%20heart%20defects%20(CHD)%20occur,the%20first%20year%20of%20life.

[2] Van Der Linde D., Konings E.E., Slager M.A., Witsenburg M., Helbing W.A., Takkenberg J.J., Roos-Hesselink J.W. (2011). Birth prevalence of congenital heart disease worldwide: A systematic review and meta-analysis. J. Am. Coll. Cardiol..

[3] Steward D.K., Ryan-Wenger N., Harrison T.M., Pridham K.F. (2020). Patterns of growth and nutrition from birth to 6 months in infants with complex congenital cardiac defects. Nurs. Res..

[4] Marino B.S., Lipkin P.H., Newburger J.W., Peacock G., Gerdes M., Gaynor J.W., Mussatto K.A., Uzark K., Goldberg C.S., Johnson W.H. (2012). Neurodevelopmental outcomes in children with congenital heart disease: Evaluation and management: A scientific statement from the American Heart Association. Circulation.

[5] Ravishankar C., Zak V., Williams I.A., Bellinger D.C., Gaynor J.W., Ghanayem N.S., Krawczeski C.D., Licht D.J., Mahony L., Newburger J.W. (2013). Association of impaired linear growth and worse neurodevelopmental outcome in infants with single ventricle physiology: A report from the pediatric heart network infant single ventricle trial. J. Pediatr..

[6] Jeffries H.E., Wells W.J., Starnes V.A., Wetzel R.C., Moromisato D.Y. (2006). Gastrointestinal morbidity after Norwood palliation for hypoplastic left heart syndrome. Ann. Thorac. Surg..

[7] Varan B., Tokel K., Yilmaz G. (1999). Malnutrition and growth failure in cyanotic and acyanotic congenital heart disease with and without pulmonary hypertension. Arch. Dis. Child..

[8] Butler S.C., Huyler K., Kaza A., Rachwal C. (2017). Filling a significant gap in the cardiac ICU: Implementation of individualised developmental care. Cardiol. Young.

[9] Ringle M.L., Wernovsky G. (2016). Functional, quality of life, and neurodevelopmental outcomes after congenital cardiac surgery. Semin. Perinatol..

[10] Medoff-Cooper B., Ravishankar C. (2013). Nutrition and growth in congenital heart disease: A challenge in children. Curr. Opin. Cardiol..

[11] Lisanti A.J., Uzark K.C., Harrison T.M., Peterson J.K., Butler S.C., Miller T.A., Allen K.Y., Miller S.P., Jones C.E. (2023). American Heart Association Pediatric Cardiovascular Nursing Committee of the Council on Cardiovascular and Stroke Nursing; Council on Lifelong Congenital Heart Disease and Heart Health in the Young; and Council on Hypertension. Developmental care for hospitalized infants with complex congenital heart disease: A science advisory from the American Heart Association. J. Am. Heart Assoc..

[12] Smith L.M., Harrison T.M. (2023). Neurodevelopment in the Congenital Heart Disease Population as Framed by the Life Course Health Development Framework. J. Cardiovasc. Nurs..

[13] Daniels J.M., Harrison T.M. (2016). A case study of the environmental experience of a hospitalized newborn infant with complex congenital heart disease. J. Cardiovasc. Nurs..

[14] Lisanti A.J., Vittner D., Medoff-Cooper B., Fogel J., Wernovsky G., Butler S. (2019). Individualized family-centered developmental care: An essential model to address the unique needs of infants with congenital heart disease. J. Cardiovasc. Nurs..

[15] Lisanti A.J., Golfenshtein N., Medoff-Cooper B. (2017). The pediatric cardiac intensive care unit parental stress model: Refinement using directed content analysis. Adv. Nurs. Sci..

[16] Tsui W.K., Yip K.H., Yip Y.C. (2023). Heartbreak and loneliness due to family separations and limited visiting during COVID-19: A qualitative study. IJERPH.

[17] Dilli D., Aydin B., Zenciroğlu A., Özyazici E., Beken S., Okumuş N. (2013). Treatment outcomes of infants with cyanotic congenital heart disease treated with synbiotics. Pediatrics.

[18] Elgersma K.M., McKechnie A.C., Gallagher T., Trebilcock A.L., Pridham K.F., Spatz D.L. (2021). Feeding infants with complex congenital heart disease: A modified Delphi survey to examine potential research and practice gaps. Cardiol. Young.

[19] Nordenström K., Lannering K., Mellander M., Elfvin A. (2020). Low risk of necrotising enterocolitis in enterally fed neonates with critical heart disease: An observational study. Arch. Dis. Child. Fetal Neonatal Ed..

[20] Newcombe J., Fry-Bowers E. (2017). A Post-operative Feeding Protocol to Improve Outcomes for Neonates with Critical Congenital Heart Disease. J. Pediatr. Nurs..

[21] Spillane N.T., Kashyap S., Bateman D., Weindler M., Krishnamurthy G. (2016). Comparison of Feeding Strategies for Infants with Hypoplastic Left Heart Syndrome: A Randomized Controlled Trial. World J. Pediatr. Congenit. Heart Surg..

[22] Slicker J., Hehir D.A., Horsley M., Monczka J., Stern K.W., Roman B., Ocampo E.C., Flanagan L., Keenan E., Lambert L.M. (2013). Nutrition algorithms for infants with hypoplastic left heart syndrome; birth through the first interstage period. Congenit. Heart Dis..

[23] Sood E., Berends M.W., Butcher J.L., Lisanti A.J., Medoff-Cooper B., Singer J., Willen E., Butler S. (2016). Developmental care in North American pediatric cardiac intensive care units: Survey of current practices. Adv. Neonat. Care..

[24] LaRonde M.P., Connor J.A., Cerrato B., Chiloyan A., Lisanti A.J. (2022). Individualized family-centered developmental care for infants with congenital heart disease in the intensive care unit. Am. J. Crit. Care..

[25] Chorna O.D., Slaughter J.C., Wang L., Stark A.R., Maitre N.L. (2014). A pacifier-activated music player with mother′s voice improves oral feeding in preterm infants. Pediatrics.

[26] Cevasco A.M., Grant R.E. (2005). Effects of the pacifier activated lullaby on weight gain of premature infants. J. Music. Ther..

[27] Standley J.M. (2000). The effect of contingent music to increase non-nutritive sucking of premature infants. Pediatr. Nurs..

[28] Standley J.M., Cassidy J., Grant R., Cevasco A., Szuch C., Nguyen J., Walworth D., Procelli D., Jarred J., Adams K. (2010). The effect of music reinforcement for non-nutritive sucking on nipple feeding of premature infants. Pediatr. Nurs..

[29] Richard C., Jeanvoine A., Stark A.R., Hague K., Kjeldsen C., Maitre N.L. (2022). Randomized Trial to Increase Speech Sound Differentiation in Infants Born Preterm. J. Pediatr..

[30] O’Brien S.M., Clarke D.R., Jacobs J.P., Jacobs M.L., Lacour-Gayet F.G., Pizarro C., Welke K.F., Maruszewski B., Tobota Z., Miller W.J. (2009). An empirically based tool for analyzing mortality associated with congenital heart surgery. J. Thorac. Cardiovasc. Surg..

[31] Jadcherla S.R., Vijayapal A.S., Leuthner S. (2009). Feeding abilities in neonates with congenital heart disease: A retrospective study. J. Perinatol..

[32] Moon C. (2017). Prenatal Experience with the Maternal Voice. Early Vocal Contact and Preterm Infant Brain Development: Bridging the Gaps between Research and Practice.

[33] DeCasper A.J., Fifer W.P. (1980). Of human bonding: Newborns prefer their mothers′ voices. Science.

[34] Lee G.Y., Kisilevsky B.S. (2014). Fetuses respond to father′s voice but prefer mother′s voice after birth. Dev. Psychobiol..

[35] Chorna O., Hamm E., Shrivastava H., Maitre N.L. (2018). Feasibility of ERP biomarker use to study effects of mother’s voice exposure on speech sound differentiation of preterm infants. Dev. Neuropsychol..

[36] Beauchemin M., Gonzalez-Frankenberger B., Tremblay J., Vannasing P., Martínez-Montes E., Belin P., Beland R., Francoeur D., Carceller A.M., Wallois F. (2011). Mother and stranger: An electrophysiological study of voice processing in newborns. Cereb. Cortex..

[37] Swanson V., Nicol H., McInnes R., Cheyne H., Mactier H., Callander E. (2012). Developing maternal self-efficacy for feeding preterm babies in the neonatal unit. Qual. Health Res..

[38] Le Bas G.A., Youssef G.J., Macdonald J.A., Rossen L., Teague S.J., Kothe E.J., McIntosh J.E., Olsson C.A., Hutchinson D.M. (2022). The role of antenatal and postnatal maternal bonding in infant development. J. Am. Acad. Child. Adolesc. Psychiatry..

